# Mesenchymal stem cells with high telomerase expression do not actively restore their chromosome arm specific telomere length pattern after exposure to ionizing radiation

**DOI:** 10.1186/1471-2199-8-49

**Published:** 2007-06-13

**Authors:** Jesper Graakjaer, Rikke Christensen, Steen Kolvraa, Nedime Serakinci

**Affiliations:** 1 Department of Clinical Genetics, Vejle County Hospital, Vejle, Denmark; 2 Department of Anatomy and Neurobiology, Institute of Medical Biology, University of Southern Denmark, Odense, Denmark; 3 Biopark Vejle, Vejle, Denmark

## Abstract

**Background:**

Previous studies have demonstrated that telomeres in somatic cells are not randomly distributed at the end of the chromosomes. We hypothesize that these chromosome arm specific differences in telomere length (the telomere length pattern) may be actively maintained. In this study we investigate the existence and maintenance of the telomere length pattern in stem cells. For this aim we studied telomere length in primary human mesenchymal stem cells (hMSC) and their telomerase-immortalised counterpart (hMSC-telo1) during extended proliferation as well as after irradiation. Telomere lengths were measured using Fluorescence In Situ Hybridization (Q-FISH).

**Results:**

A telomere length pattern was found to exist in primary hMSC's as well as in hMSC-telo1. This pattern is similar to what was previously found in lymphocytes and fibroblasts. The cells were then exposed to a high dose of ionizing radiation. Irradiation caused profound changes in chromosome specific telomere lengths, effectively destroying the telomere length pattern. Following long term culturing after irradiation, a telomere length pattern was found to re-emerge. However, the new telomere length pattern did not resemble the telomere length pattern observed before irradiation.

**Conclusion:**

Our findings indicate that a telomere length pattern does exist in mesenchymal stem cells and that the pattern is not actively re-established after destruction by irradiation.

## Background

Telomeres consist of repetitive non-coding sequences located at the very end of all chromosomes in higher organisms [1]. The telomeres form a loop structure which in collaboration with a number of specific telomere associated proteins, protects chromosome ends from degradation and chromosome fusion [2]. Telomeres have been found to shorten with each cell division due to a process called the "end replication problem" and possibly due to acquired oxidative damage [3,4]. This gradual shortening of the telomeres during life continues until the telomeres reach a certain length, at which stage the presence of critically short telomeres triggers a p53/Rb mediated senescence pathway. Cell culture experiments show that due to this limitation of division potential, normal human cells can only divide 50–100 times [5]. It has been proposed that this limitation of division potential may limit the ultimate lifespan of human individuals [6]. An increasing body of evidence suggests that the shortest telomere in a cell is responsible for triggering the p53/Rb pathway. It is therefore highly relevant that we and others have found that telomere lengths are not randomly distributed at chromosome ends [7,8]. In a series of investigations we have thus found that two samples taken from the same individual and analyzed for chromosome specific telomere lengths, exhibit rather similar results with correlations between the two samples in the order of 0.8–0.9 (p < 0,001 always). When samples from two unrelated individuals were compared in the same way, correlations in the range of 0.3–0.85 (p < 0.001 always) were obtained [7]. These rather high correlations mean that a length pattern exists in man, and consequently that certain chromosome arms have consistently shorter telomeres than others and may therefore be prone to activation of the p53/Rb pathway. So far, this non-random distribution of telomere lengths on the chromosome arms, proposedly named "the telomere length pattern" has been found in human lymphocytes, fibroblasts and amnion cells and in individuals of all ages up to an age of 80 years, where the pattern begins to deteriorate [7-9].

Our previous studies of the telomere length pattern in monozygotic twins and in patients with constitutional translocations indicate that chromosome arm specific differences in telomere length might be actively maintained by factors located distally on the chromosome arm [7]. This is supported by recent studies of telomere length in Tetrahymena which demonstrated that telomere length is influenced by the subtelomeric regions [10].

**Figure 1:**
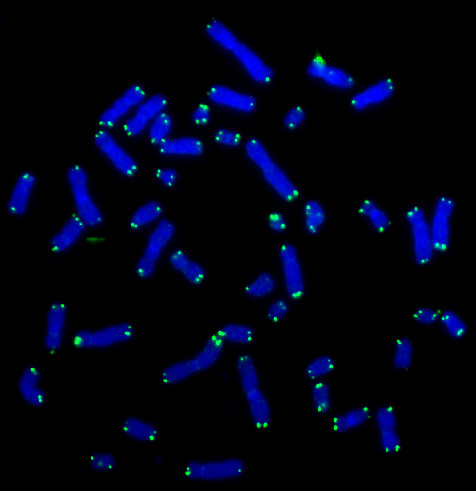
Image of chromosomes from an hTERT-telo1 cell stained with the telomere PNA probe. Telomeres are stained with FITC and chromosomes are stained with DAPI.

In this study the Q-FISH method was used to investigate the telomere length pattern in human mesenchymal stem cells (hMSC) derived from bone marrow stromal cells (Figure 1). Stem cells are unique in their ability to differentiate and self renew and may play an important role regarding telomere dynamics, since the status of the telomeres in the stem cells determines the status of the telomeres in the differentiated cells originating from them. Lately there has been an increasing interest in the potential to use adult stem cells in cell replacement strategies and in tissue engineering. Due to the simplicity of isolation, mesenchymal stem cells are one of the first to be considered for use in therapeutic applications. The hMSC's have the ability, both in vivo and in vitro, to differentiate into a variety of adult mesenchymal tissues, such as bone, cartilage, adipose and muscle. Furthermore, many of these tissues of mesenchymal origin can give rise to cancer. Therefore these cells represent a good model system for studying the chromosome arm specific telomere length pattern in stem cells.

In our investigations, we have used primary telomerase negative hMSC's and a cell line that ectopically express telomerase (hMSC-telo1). The ectopic expression was achieved by viral transduction of the cells with the catalytic subunit of telomerase hTERT [11].

## Results

### A stable telomere length pattern exists in hMSC's

**Figure 2:**
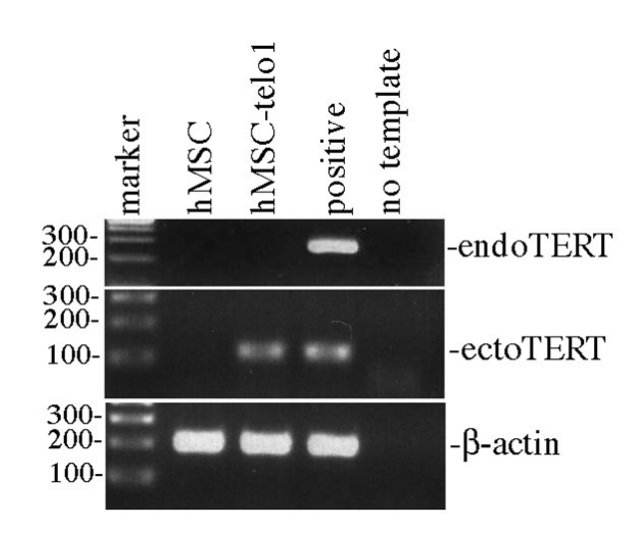
**Top panel: **Endogenous hTERT expression was not detected in hMSC and hMSC-telo1 cells. Positive control is a MG63 cell which is known to express hTERT. **Middle panel: **Ectopic hTERT was detected in hMSC-telo1 cells but not in hMSC's. Positive control is TERT transduced cells. **Bottom panel: **β-actin was found in both hMSC's and hMSC-telo1 cells. Positive control is MG63 cells.

**Figure 3:**
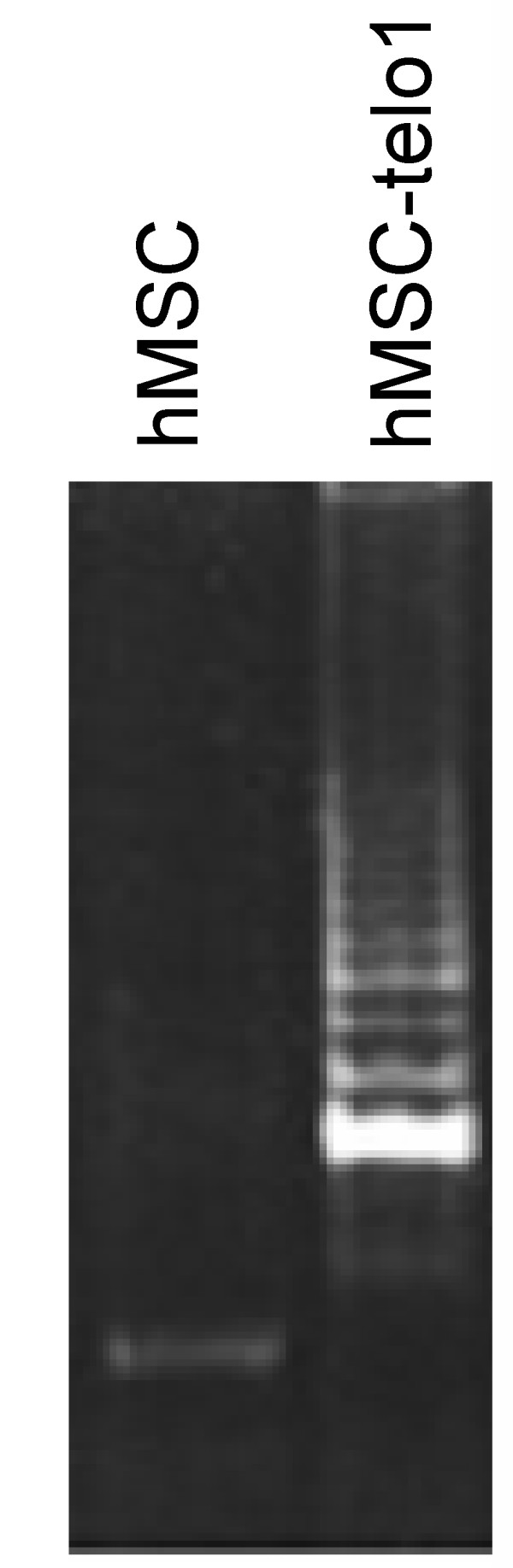
TRAP-assay confirmed a high level of telomerase activity in telomerase positive hMSC-telo1 cells while no telomerase activity was found in hMSC's.

Primary hMSC's were first tested for the presence of the endogenous hTERT gene using RT-PCR and the presence of telomerase activity using the telomeric repeat amplification protocol (TRAP) assay. Neither endogenous hTERT gene expression (Figure 2 top panel) nor telomerase activity (Figure 3) could be found.

**Figure 4:**
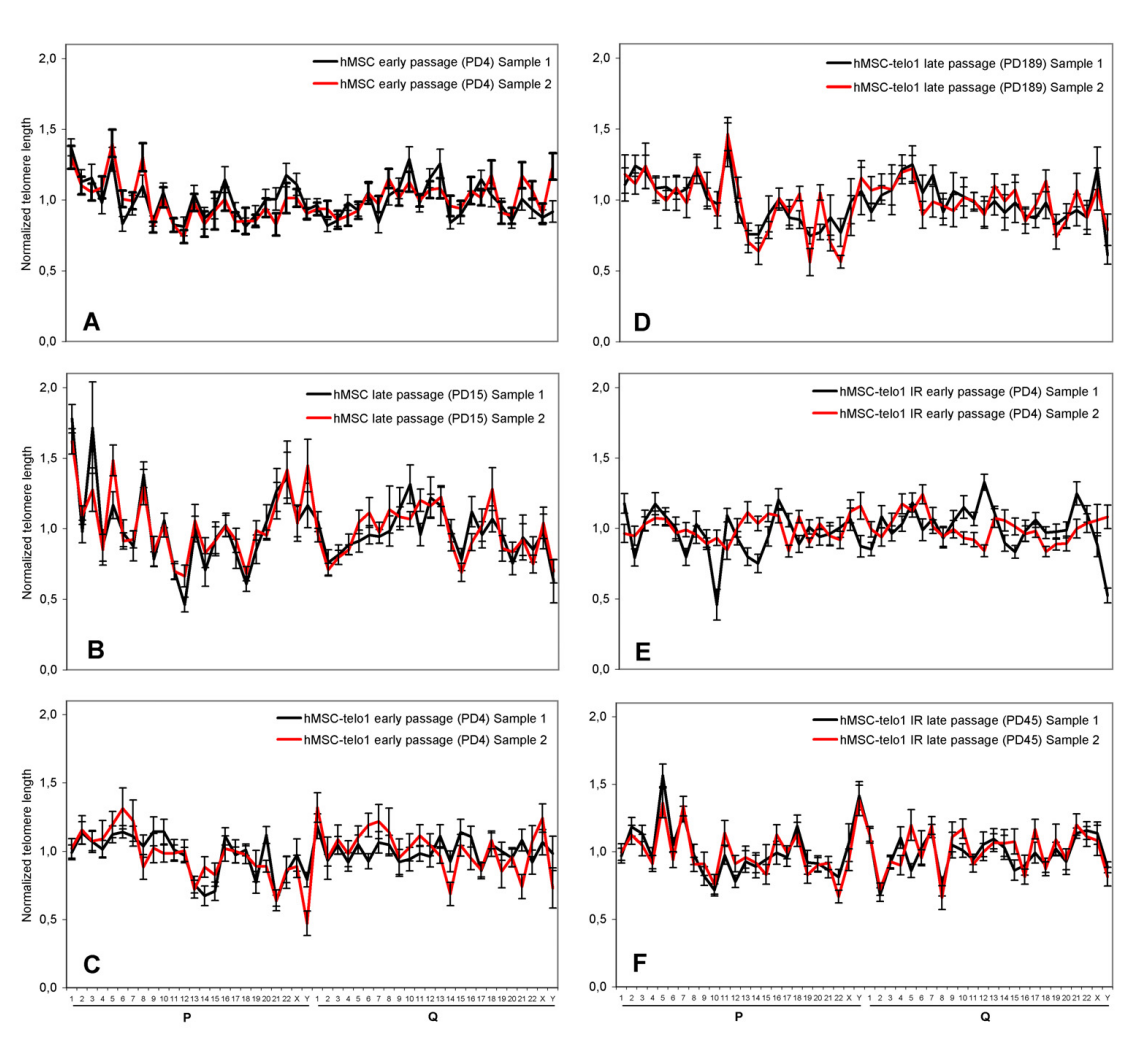
Telomere profiles. In each figure two double samples are compared. X-axis describes the chromosome arm no. and the Y-axis describes the corresponding telomere length for sample 1 (black) and sample 2 (red). Errorbars illustrates S.E.M. Each figure corresponds to one of the comparisons done in table 1. **A **Comparison between two samples from hMSC early passage. **B **Comparison between two samples from hMSC late passage. **C **Comparison between two samples from hMSC-telo1 early passage. **D **Comparison between two samples from hMSC-telo1 late passage. **E **Comparison between two samples from hMSC-telo1 irradiated early passage. **F **Comparison between two samples from hMSC-telo1 irradiated late passage.

**Figure 5:**
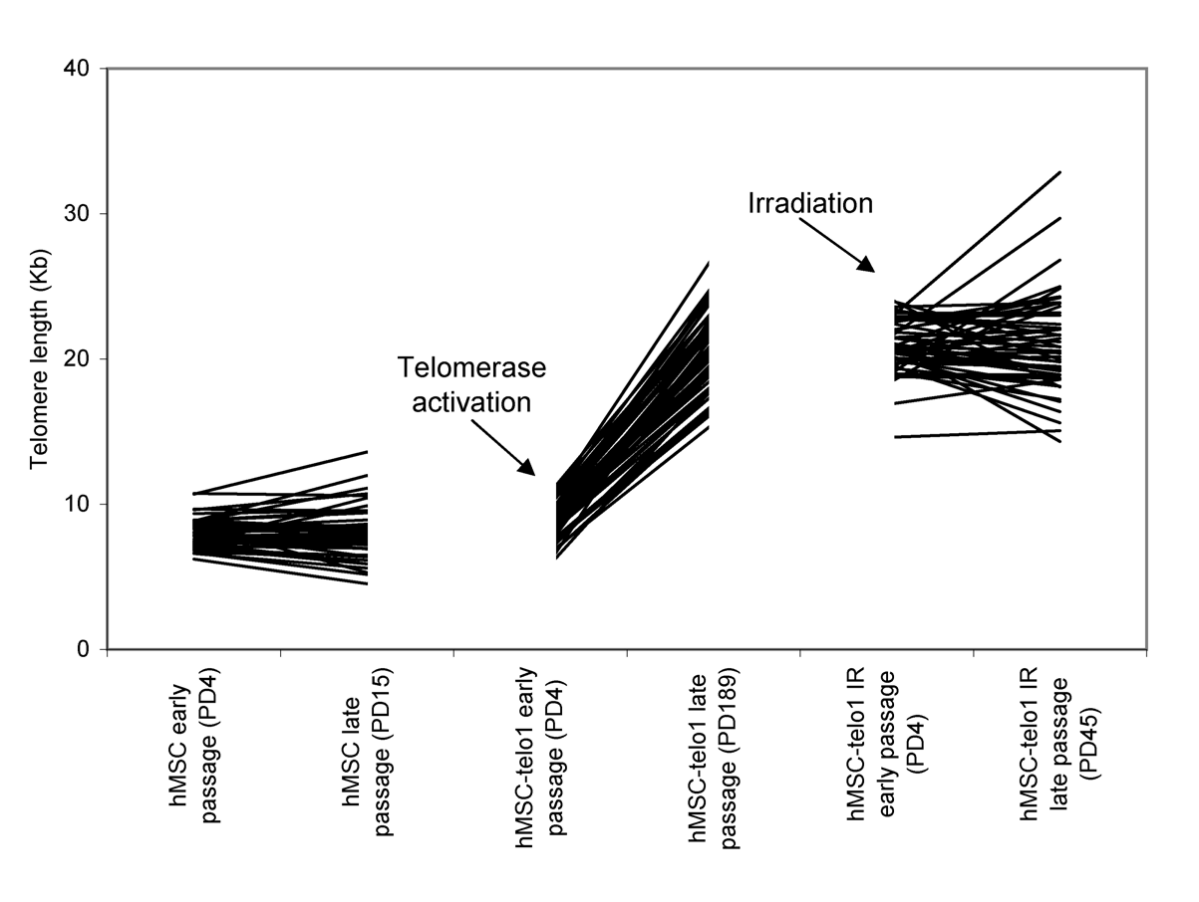
Length variation of individual chromosome ends during telomere erosion (A), telomerase mediated telomere elongation (B) and after irradiation (C). Each line represents telomere length on one chromosome end. X-axis is not to scale. Corresponding correlations can be found in table 2.

hMSC's were then tested for the presence of chromosome arm specific differences in telomere lengths. The hMSC's were grown until senescence at app. PD 22. Two samples from an early passage (PD 4) and two samples from a late passage (PD 15; last passage with sufficient amount of metaphases for analysis) were obtained. Each sample was grown separately in a slide-flask for 1–2 population doublings and then used for telomere length analysis. A significant correlation was found when comparing the telomere length pattern from two samples from the same passage which confirmed that a stable telomere length pattern was present both at early and late passage (Table 1, Figure 4A and 4B). hMSC's at early passage were then compared to hMSC's at late passage (Table 2). A correlation of 0.74 was obtained indicating that no drastic changes in the telomere length pattern appeared during this growth phase, again supporting the existence of a stable telomere pattern during the life span of the cells. TRF-gel analysis of the overall telomere length in the hMSC's showed an estimated telomere shortening rate of app. 50 bp/PD (data shown in: Serakinci et. al. Exp. Cell Res. 2007 [11]). We also calculated mean length of telomeres on the individual chromosome ends and plotted these against population doublings. The changes in individual telomere length during 11 PD's are shown in figure 5A. Although a few telomere ends break the pattern it is clearly seen that most telomeres lose length at a similar rate.

**Table 1:** Comparison of the telomere length pattern between two samples from the same passage

**Sample 1 Cell Type**	**Sample 1 Passage Stage**	**Sample 1 PD no.**	**Sample 2 Cell Type**	**Sample 2 Passage Stage**	**Sample 2 PD no.**	**Illustrated in figure no.**	**Correlation**	**p-value**
hMSC	early	4	hMSC	early	4	4A	0.69	< 0.000
hMSC	late	15	hMSC	late	15	4B	0.83	< 0.000
hMSC-telo1	early	4	hMSC-telo1	early	4	4C	0.61	< 0.000
hMSC-telo1	late	189	hMSC-telo1	late	189	4D	0.78	< 0.000
hMSC-telo1 irradiated	early	4	hMSC-telo1 irradiated	early	4	4E	-0.10	0.750
hMSC-telo1 irradiated	late	45	hMSC-telo1 irradiated	late	45	4F	0.80	< 0.000

**Table 2:** Comparison of the telomere length pattern before and after transduction and before and after irradiation

**Sample 1 Cell Type**	**Sample 1 Passage Stage**	**Sample 1 PD no.**	**Sample 2 Cell Type**	**Sample 2 Passage Stage**	**Sample 2 PD no.**	**Illustrated in figure no.**	**Correlation**	**p-value**
hMSC	early	4	hMSC	late	15	5A	0.74	< 0.000
**hMSC**			**hMSC-telo1**			6A	0.60	< 0.000
hMSC-telo1	early	4	hMSC-telo1	late	189	5B	0.49	< 0.000
**hMSC-telo1**			**hMSC-telo1 irradiated**			6B	0.15	0.1544
hMSC-telo1 irradiated	early	4	hMSC-telo1 irradiated	late	45	5C	0.30	0.011

hMSC-telo1 irradiated	late	45	hMSC-telo1				0.33	0.010

We therefore conclude that a stable telomere length pattern exists in hMSC's and that this pattern – most likely due to a similar rate of shortening on all chromosomes – is similar at both early and late phases of cellular expansion. These observations are in accordance with previous results, also indicating that telomere erosion in general is very similar on each chromosome end [9].

### A stable telomere length pattern exists in immortal hMSC's

Although no apparent deterioration of the telomere length pattern could be found in primary hMSC's, these cells only have a limited growth potential and are only cultured for a limited period of time, which may not have been adequate to induce substantial changes in the telomere length pattern. We therefore decided to study the dynamics of the telomere length pattern in telomerase immortalised hMSC's (hMSC-telo1). These cells were tested for the presence of both the endogenous and ectopic hTERT gene by RT-PCR. Only ectopic hTERT gene expression was detected (Figure 2 middle panel). TRAP assay analysis showed a high level of telomerase activity in hMSC-telo1 (Figure 3). TRF-gel analysis of total telomere length in the hMSC-telo1 cell line showed that the introduction of telomerase resulted in elongation of telomeres from a mean value of 8 kb to a mean value of 21 kb followed by a state of telomere length equilibrium after app. 43 PD's (data shown in: Serakinci et. al. Exp. Cell Res. 2007 [11]).

The activation of telomerase activity extended the life span of the mesenchymal stem cells without causing changes in stem cell characteristics and genomic stability. Analysis of the chromosomes up to 189 PD's after transduction showed a normal karyotype and morphologically the cells appeared to be small and spindle-shaped and without any evidence of senescence. In order to detect the presence of senescent cells in the hMSC-telo1 cell line, we employed staining for senescence associated Beta-gal protein [12]. Only a small and stable proportion (around 5%) of the cells stained positive for Beta-gal, confirming the maintenance of a young phenotype in spite of the extensive proliferation.

**Figure 6:**
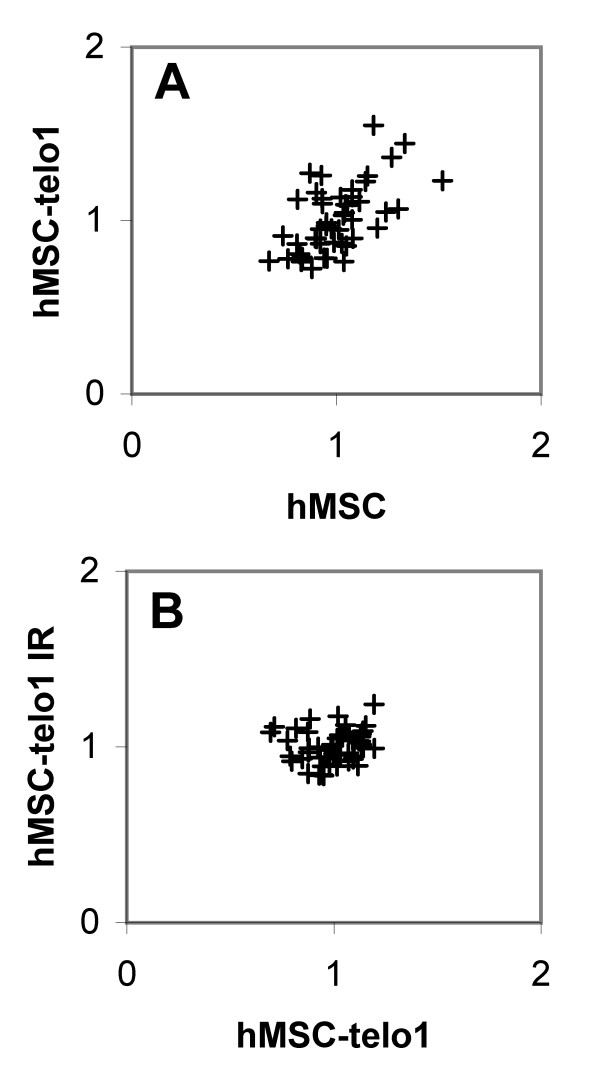
Comparison of chromosome arm specific differences in telomere length. Each dot represents the telomere length from one chromosome arm from one cell sample matched to the same chromosome arm from a second cell sample. **6A **X-axis: hMSC before transduction. Y-axis: hMSC-telo1 after transduction. **6B **X-axis: hMSC-telo1 before irradiation. Y-axis: hMSC-telo1 after irradiation with 2.5 Gy. Corresponding correlations can be found in table 2.

The telomerase positive hMSC-telo1 cells were subsequently grown for up to 189 PD and at four different time points cell samples were obtained (PD 4, PD 66, PD 180, PD 189). To investigate the immediate impact of telomerase activation on the telomere length pattern, early passage hMSC-telo1 (PD 4) was compared to hMSC's before transduction. A significant correlation of 0.60 was obtained (Table 2, Figure 6A), indicating that the telomere length pattern is largely similar before and immediately after the introduction of telomerase activity. We then investigated if a telomere length pattern existed at all mentioned measuring points during the 189 PD growth phase. A high correlation was found when comparing the telomere length pattern from two samples from the same passage (R > 0.61 Always; Early and late passage: Table 1, Figure 4C and 4D) indicating the presence of a telomere length pattern throughout the 189 PD cell culturing period. Furthermore, a significant correlation (0.49) was obtained when comparing the telomere length pattern from hMSC-telo1 cells at early passage to late passage hMSC-telo1 cells (Table 2), indicating that the telomere length pattern did not change significantly during the telomerase dependent extended life span of hMSC-telo1 cells. This was subsequently confirmed by comparing all possible combinations of samples at different time points during the growth phase. In all comparisons of the telomere length pattern at different time points, a high and significant correlation was obtained (mean R = 0.54; min. R = 0.49).

We therefore conclude that a stable telomere length pattern also exists in telomerase immortalised hMSC's and that this pattern is similar at both early and late phases of cellular expansion. It is thereby indicated that at least under the circumstances described here, the magnitude of telomerase mediated elongation is not very variable over the chromosome arms, as also illustrated in figure 5B.

### Telomerase is not able to re-establish the original telomere length pattern when it has been eradicated by irradiation

Our results indicate that the dynamics of telomere erosion/telomerase activity still allow the maintenance of a particular telomere length pattern for many PD's. This can simply be due to a highly similar telomere length alteration taking place on all chromosomes, but it can also be imagined that the striking conservation of length pattern is due to telomerase actively establishing and maintaining this specific length pattern. We and others have previously shown that irradiation of cells with ionizing radiation induces changes in the intracellular distribution of telomere lengths [11,13]. We therefore decided to investigate the possibility that telomerase plays an active role in establishing and maintaining a specific telomere length pattern by irradiating hMSC-telo1 cells with ionizing radiation and then investigating if telomerase is capable of re-establishing a telomere length pattern in the cells during a growth period following irradiation.

hMSC-telo1 cells were therefore subjected to 2.5 Gy of ionizing radiation, a dose that resulted in short term cellular growth arrest. Subtelomeric staining and chromosomal analysis of hMSC-telo1 cells that returned to cell cycling showed no significant increase in chromosomal aberrations (not shown). Following irradiation, two independent samples of hMSC-telo1 cells were then investigated at early passage after return to cell cycling using Q-FISH telomere length analysis. No significant correlation (-0.1) could be found when comparing the telomere length pattern from two different samples from the same passage (Table 1 and Figure 4E), indicating that cells from the same passage do not any longer share the same telomere length pattern. Irradiated hMSC-telo1 cells at early passages after irradiation were also compared to hMSC-telo1 cells before irradiation. Also here no significant correlation (0.15) was found (Table 2 and Figure 6B), indicating that the telomere length pattern evident in hMSC-telo1 cells, is no longer present.

Irradiated hMSC-telo1 cells were then grown for another 45 PD. Again, two samples were obtained and used for telomere length analysis. A high correlation (0.80) between the two samples from the same passage was now obtained (Table 1 and Figure 4F). This indicates that even though irradiated hMSC-telo1 cells did not share a similar telomere length pattern right after irradiation, the long term growth resulted in the occurrence of a new telomere length pattern. The new telomere length pattern did however not strongly resemble the telomere length pattern present before irradiation (correlation between hMSC-telo1 irradiated PD45 and hMSC-telo1 = 0.33; Table 2), and it also did not strongly resemble the telomere length pattern at early passage after irradiation (correlation between hMSC-telo1 irradiated PD45 and hMSC-telo1 irradiated PD4 = 0.30; Table 2). The alteration of the telomere length pattern following irradiation is also illustrated in figure 5C. Similar to earlier observations [13], no significant changes in total telomere length were observed post irradiation by TRF-gel analysis of hMSC-telo1 cells (data not shown).

## Discussion

We have in the present communication investigated the existence, creation and maintenance of the specific telomere length pattern on single chromosome arms in telomerase negative primary mesenchymal stem cells and their counterpart exhibiting high ectopic expression of the hTERT gene. It should be mentioned that telomerase status has been found to vary in primary stem cells. It can therefore not be completely ruled out that the hMSC's investigated in this study might express telomerase in some conditions. Nevertheless, similarly to what others have found [14], we do not find any endogenous telomerase expression in the primary hMSC's. On the opposite, the hMSC-telo1 cells have a high overexpression of ectopic hTERT. The two cells lines thereby represent a unique opportunity to investigate the effect of high constitutive levels of telomerase activity on telomere length dynamics.

First of all we find that the hMSC's have a telomere length pattern. To our knowledge, this is the first observation of a telomere length pattern in non-differentiated cells. The telomere length pattern found in hMSC's has some resemblance to the telomere length pattern found in other human cell types (hMSC:fibroblast R = 0.42 p = 0.001; hMSC:lymphocytes R = 0.57 p < 0.0001) [7], and it is therefore likely that the chromosome arm specific telomere length variations found in differentiated cells may originate from telomere length variations in stem cells.

Furthermore we find that this telomere length pattern is remarkably stable in both primary and immortalised hMSC's: The telomere length pattern that we find at the end of cell culturing is essentially the same as at the beginning of cell culturing. These observations are in concordance with our previous studies of lymphocytes from aged individuals where we also find a remarkably stable telomere length pattern [7]. For the primary hMSC's we can however not rule out that changes in the telomere length pattern may occur beyond PD 15 which was the last cell sample we could reliably measure. This is due to limitations of the Q-FISH method which requires a fair number of high quality metaphases (min. 20 per sample) for reliable measurement. It should also be mentioned that non-dividing and/or senescent cells may have a different distribution of telomere lengths over the chromosomes since we can only measure dividing cells by Q-FISH. Nevertheless, we observe no apparent deterioration of the telomere length pattern during the cell culturing of the hMSC's.

Furthermore, we investigate the dynamics of the telomere length pattern during culturing of hMSC's immortalised by telomerase. First of all this allowed a cellular expansion on a much higher scale than for the primary hMSC's, but additionally we also wanted to investigate the effect of high telomerase expression on the telomere length pattern. However, although an increase in overall telomere length was observed, neither the activation of telomerase nor a 189 PD growth period caused any drastic changes in the telomere length pattern.

It should be noted that these results diverge from a previous study where a homogenization of telomere lengths was found following the introduction of exogenous telomerase [15]. A homogenization of telomere lengths following telomerase activation is in accordance with a preferential elongation of the critically short telomeres in the cell, while our results indicate the existence of a separate mechanism maintaining a specific pattern. The two mechanisms do not exclude each other and it is possible that the activity depends on the type and status of the cell and may therefore simply be explained by the fact that our study was done in human stem cells while the previous study was done in human fibroblast cells. Another possibility, however, is that the divergent results are due to different levels of telomerase activity. In this context a recently proposed model describing telomere length homeostasis suggests that all telomeres are accessible to telomerase for a short period of time after DNA replication, but long telomeres switch back more quickly to a non-extendible state and therefore short telomeres may have a higher probability to bind telomerase [16]. At low telomerase concentration levels, therefore, only short telomeres are elongated, but at high telomerase concentration, where the probability of telomerase binding is high, all telomeres will be extended. In the present communication we are investigating cells with a high level of telomerase activity and therefore obtain a substantial increase in average telomere length (from 8 kb to 21 kb) following telomerase activation. In contrast, Londono-Vallejo et al. observe only a minor increase in telomere length from 7.3 to 8 kb following telomerase activation suggesting that telomerase activity levels may be markedly different in the two studies. It is conceivable that our study primarily represents telomere dynamics in high telomerase activity conditions while the previous study primarily represents telomere dynamics during low telomerase activity conditions.

Considering the amount of cell divisions required for the cellular expansion that we observe during the 189 PD growth period of the hMSC-telo1 cells, it is quite remarkable that no drastic changes in the telomere length pattern is observed. We estimate that at 189 PD, the immortalised hMSC's would have been subjected to a total telomere shortening of app. 9.5 kbp. This estimate is based on our observation of a shortening rate of app. 50 bp/cell division in primary hMSC's, which is in line with other observations of telomere shortening rate in other human cells (50–150 bp/cell division) [17-19]. Since the cells at 189 PD are at a stable telomere length equilibrium of 21 kbp, telomerase mediated telomere elongation from 8 to 21 kbp has not only compensated for the 9.5 kbp telomere loss, but also added an additional 13 kbp to the telomeres. The total amount of expected telomere elongation therefore reaches app. 22.5 kbp, which is more than the total length of the telomeres at equilibrium (21 kbp). It is therefore conceivable that at 189 PD, the cells will have passed through a considerable amount of shortening/elongation cycles. Conservation of a length pattern in spite of such an extensive telomere synthesis means that either telomere erosion or telomerase-catalyzed elongation, or both, is highly regulated, resulting in exactly the same loss/gain of telomere sequences on all chromosomes. Indirect support for this, is lent by the fact that we have previously demonstrated a very similar relative telomere length on the same chromosome present in two aged MZ twins [9]. In a separate investigation we have found that telomere lengths on chromosomes with constitutional distal translocations in all investigated cases resembled the normal telomere length on the donor chromosome arm and did not resemble the normal length of the recipient chromosome arm [7]. This led us to speculate that chromosome arm specific differences in telomere lengths might be actively maintained by a cis-acting telomere length influencing/-determining factor located distally on the chromosome arm. One possible mechanism whereby such a distal factor might influence individual telomere length could be through differences in accessibility of telomerase to the different telomeres. In this regard, recent evidence suggests that subtelomeric variations in DNA and histone methylation may regulate telomere length by varying the accessibility of telomerase to the telomeres [20,21].

In the present study we have further addressed the possibility that the telomere length pattern is actively maintained. This was done by removing the common telomere length pattern by irradiation and subsequently investigating if the common length pattern was re-established by the action of telomerase. The overall results of these irradiation series are compatible with a scenario where irradiation immediately destroys the length pattern in a random manner, resulting in loss of similarity between the length patterns of different individual cells. Subsequent, prolonged culture resulted in the re-appearance of a telomere length pattern in the culture. However, since the pattern that re-emerged after irradiation did not resemble the pattern existing before irradiation, our current results do not support the existence of a distally located factor that influences telomere length on the single chromosome arm. Instead the appearance of a different telomere length pattern is more likely explained by an increased clonality of the culture caused by the varying cell viability following irradiation and the eventual overgrowth of the most viable cells. If this is the case, then the overall conclusion must be that telomerase faithfully preserves a length distribution that is present in a cell, but do not create a new pattern or modify it in a specific way.

## Conclusion

The mechanism that leads to a common telomere length pattern is still unknown. One – rather unlikely – possibility is that the profile is ancient and conserved in man only due to a very precise telomere shortening/elongation on all chromosome ends. If this is not the case, then one must assume that either the telomere shortening or the telomerase catalyzed telomere elongation at some point acts differentially on different chromosome ends, thereby re-establishing the length pattern. We find it unlikely that it is the dynamics of the telomere shortening that establish the length pattern, simply because we have found that in very old individuals the pattern starts to deteriorate [7]. In regards to telomerase-catalyzed elongation, then the results in the present communication suggest that highly active telomerase does not by itself have a differential effect on chromosome ends in stem cells. It may, however, be that the germ cells have a special telomere structure leading to differential elongation in these cells only, resulting in re-establishment of the length pattern in connection with gametogenesis. In order to substantiate this hypothesis we have initiated studies on telomere length patterns in the various stages of germ cell maturation.

## Methods

### Cell culture and transduction of hTERT

The mesenchymal stem cells were isolated from bone marrow by centrifugation (700 g for 15 minutes at 4°C) over a Ficoll Hypaque gradient (Sigma-Aldrich, St. Louis, MO, USA) [22]. hTERT was introduced using a retroviral transduction system [11]. Normal and immortal hMSC's were cultivated in high glucose (4.5 g/l) Dulbeco's modified Eagles medium (DMEM, Gibco, Life Technology, Rockville, MD, USA) supplemented with 10% fetal bovine serum (Gibco, Life technology), 100 U/ml of penicillin and streptomycin (Gibco, Life technology) and 2 mM of L-glutamine. All cells were maintained in a humidified incubator at 37°C and 5% CO_2_.

### Telomerase expression assay

RNA was isolated from cells using the High Pure RNA Tissue Kit (Roche, Hvidovre, Denmark). cDNA was synthesized from RNA using the 1^st ^Strand cDNA Synthesis Kit for RT-PCR (AMV) (Roche) according to manufacturer's instructions. β-actin primers were used in a parallel reaction as a control of the cDNA preparation and for detection of possible DNA contamination in PCR reactions that were performed on RNA.

The following hTERT-gene primers were used: endogenous TERT:sense: 5'-CTG CTG CGC ACG TGG GAA GC-3' antisense: 5'-GGA CAC CTG GCG GAA GGA G-3' ectopic hTERT: sense:5'-GGA CCA TCT CTA GAC TGA CG-3 antisense:5'-GGA GCG CAC GGC TCG GCA GC-3'

*RT-PCR conditions for endogenous TERT*: After initial denaturation at 95°C for 15 min, amplification was performed for 40 cycles of 94°C for 30 s, 60°C for 30s, 72°C for 1 min, and finally 72°C for 10 min. *RT-PCR conditions for ectopic-hTERT: *After an initial denaturation step at 94°C for 3 min, amplification was performed for 30 cycles at 94°C for 30 s, 59°C for 30 s and 72°C for 1 min, followed by a final extension step at 72°C for 10 min.

### Telomerase activity assay

Telomerase activity was tested using a TRAP assay kit: TeloTAGGG Telomerase PCR ELISA kit (Roche).

### Cell proliferation studies

Cells were expanded by consecutive subcultivations in DMEM with 10% FCS. Long-term cell growth in vitro was determined by calculating population doubling level (PD). The initial seeding number (Nstart) and the 80% confluence harvested cell number (Nfinish) was used to calculate the population doubling level PD = ln(N-Finish/N-Start)/ln2. Thus, cumulative population doubling level is the sum of PD.

### Beta-galactosidase assay

Cells were incubated at 37°C (no CO_2_) overnight with fresh Beta-gal. solution containing 1 mg of 5-bromo-4-chloro-3-indolyl B-D-galactoside (X-Gal, Sigma) per ml, 40 mM citric acid (pH 6.0), 5 mM potassium ferrocyanide, 5 mM potassium ferricyanide, 150 mM NaCl, 2 mM MgCl_2_. From each slide flask, minimum of 500 cells were counted under the microscope. The experiment was repeated 3 times and the mean percent of Beta-galactosidase-positive cells was then calculated.

### Irradiation of cells

Cells were irradiated using a Gammacelle 2000 RH^137^Cs irradiator (AEK, Riso, Denmark). Irradiation levels were 2.5 Gy delivered at a rate of 2.5 Gy/min. The culture medium was changed immediately after irradiation.

### Telomere restriction fragment (TRF) analysis

Telomere lengths were determined using the *T*elo*TAGGG *Telomere Length Assay (Roche). Briefly, three μg of genomic DNA was digested with the restriction enzymes *Hin*fI and *Rsa*I and analysed by Southern blotting using a digoxenin (DIG)-labelled TTAGGG probe. Fragment distribution was detected after incubation with a DIG-specific antibody covalently coupled to alkaline phosphatase and a chemiluminescence substrate. The average TRF lengths were determined by comparing the fragment distribution to a series of molecular weight standards.

### Chromosome analysis

Prior to chromosome and FISH analysis, cells were grown in slideflasks for 2–3 days. Cells were then treated with colcemide (10 μg/μl) for 30 min. followed by hypotonic shock (60 mM KCl) and methanol/acetic acid fixation.

### Telomere-QFISH

#### Telomere staining

A telomere FISH kit (DAKO, Glostrup, Denmark) was used for telomere staining (Figure 1). The probe used was a FITC conjugated (CCCTAA)3 PNA probe. The staining was applied according to manufacturer. For subsequent karyotyping, the DNA was then stained with a DAPI solution (0.5 μg/ml) also containing antifade (0.11 % phenylenediamine dihydrochloride).

#### Image acquisition and analysis

Fluorescent specific signals were visualized by fluorescence microscopy and captured at 100 × magnification with a cooled CCD camera using IpLab software. Standard DAPI and FITC filters were used. Telomere images were acquired using a constant exposure time (10 sec.) throughout the study. For each sample, 20 digital images of metaphases were acquired and stored for analysis. Images were karyotyped using Quips CGH/karyotyper. Telomere intensity was measured using Telomere Quantifier v. 1.0 [7].

#### Normalisation of telomere length values

Telomere length values were normalised for each metaphase using the following equation:

Z = X/μ

Where:

Z = Normalised telomere length value

X = raw telomere length value

μ = mean telomere length of all telomeres in the metaphase

The final telomere length estimate for a chromosome arm was calculated by averaging normalised telomere length values from 20 metaphases.

The normalisation procedure causes loss of information regarding biological differences in the overall telomere length between cells and samples. In return for this loss, sources of variation such as daily fluctuations in UV-lamp intensity and variation in hybridisation efficiency over the slide are effectively eliminated. In this study we have therefore chosen to use normalised telomere length values for estimation of chromosome arm specific variations in telomere length and TRF analysis for estimation of absolute telomere lengths. An estimate of the absolute telomere length of a given chromosome arm can then be obtained by multiplying the relative telomere length of that chromosome arm with the estimated mean telomere length of the cell population investigated. The rationale and normalisation procedure is more extensively described in: Pommier *et. al*. Methods Mol. Bio. 2002; Graakjaer *et. al*. Mech. Ageing. Dev. 2003 [7,23].

### Statistical analysis

Pearson correlations were calculated. p-values for the correlation were calculated using TuesT-software. ANOVA analysis was performed using 95% Confidence interval.

## Authors' contributions

JG carried out Q-FISH experiments, image acquisition and analysis, data analysis and statistical analysis and drafted the manuscript. RC carried out part of the cell culturing, expression assays, telomere length assays and irradiation and helped with drafting the manuscript. SK participated in the design and coordination of the study, helped conceive the study and helped with data analysis and drafting the manuscript. NS conceived and coordinated the study, and carried out cell culturing, transduction, expression and activity assays, helped with telomere length assays and irradiation, did the cell proliferation studies, helped with data analysis and drafting of the manuscript.
